# Clinical Evaluation of BioFire COVID-19 Test, BioFire Respiratory Panel 2.1, and Cepheid Xpert Xpress SARS-CoV-2 Assays for Sample-to-Answer Detection of SARS-CoV-2

**DOI:** 10.3390/genes14010233

**Published:** 2023-01-16

**Authors:** Joonhong Park, So Yeon Kim, Jaehyeon Lee, Ki Ho Hong

**Affiliations:** 1 Department of Laboratory Medicine, Jeonbuk National University Medical School and Hospital, Jeonju 54907, Republic of Korea; 2 Research Institute of Clinical Medicine of Jeonbuk National University-Biomedical Research Institute of Jeonbuk National University Hospital, Jeonju 54907, Republic of Korea; 3 Department of Laboratory Medicine, National Medical Center, Seoul 04564, Republic of Korea; 4 Department of Laboratory Medicine, Yonsei University College of Medicine, Seoul 03722, Republic of Korea

**Keywords:** sample-to-answer RT-PCR, next-generation diagnostic systems, SARS-CoV-2, COVID-19, BioFire COVID-19 Test, BioFire Respiratory Panel 2.1, Xpert Xpress SARS-CoV-2

## Abstract

Background: Due to the extreme infectivity of SARS-CoV-2, sample-to-answer SARS-CoV-2 reverse transcription (RT) polymerase chain reaction (PCR) assays are urgently needed in order to facilitate infectious disease surveillance and control. The purpose of this study was to evaluate three sample-to-answer SARS-CoV-2 RT-PCR assays—BioFire COVID-19 Test, BioFire RP 2.1, and Cepheid Xpert Xpress SARS-CoV-2—using clinical samples. Methods: A total of 77 leftover nasopharyngeal swab (NP) swabs (36 positives and 41 negatives) confirmed by reference SARS-CoV-2 RT real-time (q) PCR assay were collected. The clinical sample concordance, as specified by their respective emergency use authorizations (EUAs), in comparison to the reference SARS-CoV-2 RT-qPCR assay, was assessed. Results: The results showed that all three sample-to-answer SARS-CoV-2 RT-PCR assays provided perfectly concordant results consistent with the reference SARS-CoV-2 RT-qPCR assay. The BioFire COVID-19 Test exhibited the best turnaround time (TAT) compared to the other assays, regardless of the test results, using one-way analysis of variance followed by Scheffe’s post hoc test (*p* < 0.001). The Xpert Xpress SARS-CoV-2 showed a shorter average TAT (mean ± standard deviation, 49.9 ± 3.1 min) in the positive samples compared to that (55.7 ± 2.5 min) of the negative samples. Conclusions: Our evaluation demonstrates that the BioFire COVID-19 Test, BioFire RP 2.1, and Cepheid Xpert Xpress SARS-CoV-2 assays compare favorably to the reference SARS-CoV-2 RT-qPCR assay, along with a 100% concordance in assay results for clinical samples and an acceptable analytical performance at their guaranteed limits of detection. The addition of a widely used simultaneous sample-to-answer SARS-CoV-2 RT-PCR assay will contribute to the number of medical laboratories able to test for COVID-19.

## 1. Introduction

Given its analytical sensitivity and specificity, the gold-standard diagnosis for SARS-CoV-2 infection is the identification of viral RNA in nasopharyngeal (NP) swabs by reverse transcription (RT) real-time (q) polymerase chain reaction (qPCR) [1,2]. Due to the very high infectivity of SARS-CoV-2, and for the purpose of infection control, sample-to-answer SARS-CoV-2 RT-PCR assays that can be employed directly from respiratory samples without multiple steps, such as nucleic acid extraction or PCR preparation, are required. In particular, the reference SARS-CoV-2 RT-qPCR assay requires several PCR processes, including RNA extraction, reagent preparation, and manual reaction assembly, as well as highly trained personnel to maintain a quality control. To improve turnaround times (TATs) and overcome supply chain obstacles and these limitations, medical institutes began relying on the use of sample-to-answer RT-PCR assays for infectious-disease surveillance and control. A substantial benefit of the application of these simultaneous assays is the reduced use of samples and reagents according to integrated sample processing [3]. To date, various sample-to-answer SARS-CoV-2 RT-PCR assays have emerged as rapid emergency use authorization (EUA) COVID-19 assay platforms that satisfy this need [4,5]. In particular, the BioFire COVID-19 Test (BioFire Defense, LLC, Salt Lake City, UT, USA) identifies three targets within the open reading frame (ORF) region of the SARS-CoV-2 genome, and the BioFire Respiratory Panel 2.1 (RP 2.1; BioFire Diagnostics, LLC, Salt Lake City, UT, USA) was targeted by specific primers for the spike (S) and membrane (M) genes of SARS-CoV-2 to the existing CE-marked and FDA-cleared BioFire RP2 test [6]. These BioFire assays are conducted on the FilmArray 2.0 and/or the FilmArray Torch Instrument systems, using endpoint DNA melting curve analysis to identify and produce a result for each target assay. On the other hand, the Cepheid Xpert Xpress SARS-CoV-2 (Xpert Xpress; Cepheid, Inc., Sunnyvale, CA, USA) is a sensitive and specific random access, cartridge-based RT-qPCR, which targets two genes, the *N2* region of the nucleocapsid (*N*) and envelope (*E*) genes [7]. As of December 2022, comparative evaluations of BioFire RP2.1 and high-throughput SARS-CoV-2 assay platforms or other sample-to-answer SARS-CoV-2 RT-PCR assay [8,9,10,11] using serial dilutions of high-titer positive samples, or reference SARS-CoV-2 RT-qPCR assay [12], have been reported [13,14].

The purpose of this study was to evaluate three sample-to-answer SARS-CoV-2 RT-PCR assays—BioFire COVID-19 Test, RP 2.1, and Cepheid Xpert Xpress—using clinical samples, and to assess the clinical sample concordance specified by their respective EUAs, in comparison to a reference SARS-CoV-2 RT-qPCR assay.

## 2. Materials and Methods

### 2.1. Sample Collection and Storage

A total of 77 residual natural NP swabs in T-SWAB TRANSPORT Universal Transport Medium (Noble Biosciences, Hwaseong, Republic of Korea) or eNAT tube (Copan Italy, Brescia, Italy) samples left over from SARS-CoV-2 molecular testing performed in symptomatic hospitalized patients or hospitalized patient of closed contacted to con-firmed case were collected between September 2020 and December 2020 at the departments of laboratory medicine of the Jeonbuk National University Hospital (Jeonju, Republic of Korea), Seoul Medical Center (Seoul, Republic of Korea), and National Medical Center (Seoul, Republic of Korea). Following routine SARS-CoV-2 molecular testing, the remaining samples were stored immediately at –80 °C in a deep freezer and then the frozen samples were transported to the Jeonbuk National University Hospital overnight on dry ice, after which they were stored at –80 °C in a deep freezer until comparative testing occurred. The patients’ medical records, such as gender, age, underlying disease, and diagnosis at the time of admission, were anonymized and were not accessible after sample collection.

### 2.2. Reference SARS-CoV-2 Reverse Transcription Real-Time PCR Assay

The reference method for our study was the standard M nCoV real-time detection method (standard M; SD Biosensor, Osong, Republic of Korea). Briefly, the reaction volume was 30 µL (20 µL of master mix and 10 µL of extracted RNA) and the standard M identifies two targets: the *RdRp* and *E* genes. A cycle threshold (Ct) value of ≤36 was defined as positive for each target gene. To discriminate false-positive or false-negative results, the SARS-CoV-2 data resulting from the standard M were reconfirmed by the Allplex SARS-CoV-2 kit (Allplex; Seegene, Seoul, Republic of Korea) as an alternative reference SARS-CoV-2 RT-qPCR assay. The Allplex targets for three SARS-CoV-2 genes; namely, the *RdRp*, *N*, and spike (*S*) genes. The RT-qPCR assay was conducted in a total volume of 20 µL (10 µL of master mix and 10 µL of extracted RNA). A Ct value of ≤ 38 was defined as positive for each target gene [15].

### 2.3. Sample-to-Answer SARS-CoV-2 Reverse Transcription PCR Assay

All samples were thawed at 4 °C and tested as close to the time of collection as possible on the day of comparative testing. All samples were initially processed in a biosafety cabinet based on the guidelines for laboratory diagnosis of COVID-19 [16]. BioFire COVID-19 Test and RP 2.1 were performed using BioFire Filmarray Torch Systems (BioFire), and Xpert Xpress was conducted using GeneXpert Instrument System (Cepheid). Sample testing with both assays was completed according to each company’s instructions for use [5]. Before comparative evaluation, the analytical accuracy, sensitivity, and specificity guaranteed by the manufacturers in three sample-to-answer SARS-CoV-2 RT-PCR assays were confirmed with assayed reference materials such as the AccuPlex SARS-CoV-2 Reference Material Kit (SeraCare, Milford, MA, USA), and spiked samples with RNA distributed from Korean National Culture Collection for Pathogens (NCCP number: 43326). Performance characteristic information of these sample-to-answer SARS-CoV-2 RT-PCR assays provided by the manufacturer are described in Table 1.

### 2.4. Statistical Analysis

Descriptive statistics were recorded as medians and interquartile ranges, or as the means ± standard deviations (SDs), as appropriate. The relationship between quantitative Ct values was estimated by means of the Bland–Altman-plot method and Passing–Bablok regression analysis. The reference method was defined as the result obtained from the standard M. Sensitivity, specificity, percentage negative agreement (NPA), percentage positive agreement (PPA), accuracy, Kappa value, and two-sided 95% confidence interval (CI) values were tested using MedCalc (version 19.5.3.; MedCalc Software, Ostend, Belgium). *p* values of <0.05 were statistically considered significant. Cohen’s kappa values (Κ) were estimated as a measure of overall agreement, with values classified as representing no results (values of 0 to 0.20), minimal (0.21 to 0.39), weak (0.40 to 0.59), moderate (0.60 to 0.79), strong (0.80 to 0.90), or almost perfect (>0.90).

## 3. Results

### 3.1. Distribution of Ct Values on Samples Analyzed on Reference SARS-CoV-2 RT-qPCR Assay

Because the three sample-to-answer SARS-CoV-2 RT-PCR assays demonstrated that low-viral-load samples could show false negatives, we estimated the distribution of the Ct values present in 36 positive samples on the standard M as the reference SARS-CoV-2 RT-qPCR assay. The presence of SARS-CoV-2 resulting from the standard M was reconfirmed by the Allplex. As a result, the Ct values ranged from 27.2 to 33.8 for the *RdRp* gene and from 27.1 to 34.3 for the *E* gene, with means of 31.4 and 30.7, respectively. In total, 58.3% (21/36) and 66.7% (24/36) of the positive samples showed Ct values below 32 for the *RdRp* and the *E* genes, respectively. When we plotted the Ct values of the *RdRp* and the *E* genes against each other in the 36 positive samples, the standard M showed a strong regression coefficient between the *E* gene specific for all of the Sarbecovirus and the *RdRp* gene for SARS-CoV-2 (y = −4.29 + 1.12 x; r = 0.882 by Spearman’s rank correlation coefficient; *p* < 0.0001), as well as a minimal delta difference between two genes (mean ± 2SD, 0.7 ± 1.7; 95% CI, 0.4 to 1.0).

### 3.2. Comparison of Sample-to-Answer SARS-CoV-2 Reverse Transcription PCR Assays

Comparative testing was conducted on 77 leftover clinical samples, and the results were assessed against those resulting from the reference method. All three sample-to-answer SARS-CoV-2 RT-PCR assays showed almost perfectly concordant results consistent with the standard M as the reference SARS-CoV-2 RT-qPCR assay. Thus, the concordance rate, analytical sensitivity, specificity, PPA, NPA, and accuracy of all three assays were all 100%, when the COVID-19 prevalence of about 55.1% from the Republic of Korea was applied. In the Republic of Korea, between 3 January 2020 and December 2022, there had been 28,534,558 confirmed cases of COVID-19 and 31,674 deaths, as reported to the World Health Organization (WHO). As of 26 October 2022, a total of 131,766,256 vaccine doses had been administered (https://covid19.who.int/region/wpro/country/kr; accessed on 2 January 2023). The diagnostic-performance statistics of the three sample-to-answer SARS-CoV-2 RT-PCR assays are summarized in Table 2.

The sensitivity performance of the sample-to-answer SARS-CoV-2 RT-PCR assays was estimated indirectly and compared to the standard M. Because the Xpert Xpress was the only assay to include the *E* gene among the three sample-to-answer SARS-CoV-2 RT-PCR assays, the analytical sensitivity of this assay was estimated indirectly, by comparing the Ct value for the gene to that of the standard M. From this comparison, the Xpert Xpress showed an inferior regression coefficient to that of the standard M (y = 14.00 + 0.50 x; r = 0.586; *p* = 0.0002). Moreover, the Ct value for the *E* gene tested by the Xpert Xpress was observed to have increased compared to that of the standard M (mean ± 2SD, 2.2 ± 5.6 ∆Ct; 95% CI, 1.3 to 3.2).

In terms of the TAT comparisons, the BioFire COVID-19 Test showed the statistically fastest mean TAT (mean ± SD, 45.8 ± 1.4 min in total TAT; 44.8 ± 1.5 min in TAT for positives; and 46.6 ± 0.7 min in TAT for negatives) compared to the other assays, regardless of the test results, when using one-way analysis of variance followed by Scheffe’s post hoc test (*p* < 0.001). Interestingly, the Xpert Xpress showed a shorter average TAT (49.9 ± 3.1 min) in the positive samples, compared to that (55.7 ± 2.5 min) of the negative samples. However, the differences in the TATs between the positive and negative samples were not evident on the BioFire COVID-19 Test or the RP 2.1.

## 4. Discussion

Sample-to-answer RT-PCR assays for SARS-CoV-2 simultaneously provide critical information for the diagnosis and management of COVID-19. These cartridge-based diagnostics allow the transfer of diagnoses to point-of-care scenarios, including smaller medical laboratories; furthermore, the rapid assessment of suspected cases, allows for specific epidemiological management [5]. In this study, to avoid pre-analytical variables associated with sample collection, the sample preparation process, the sample storage conditions, and reagent thawing, which may have influenced the length of the laboratory-personnel time and altered the SARS-CoV-2 results, a comparative evaluation was conducted as close to the time of collection as possible in the same testing environment by shipping the samples to the medical laboratory in Jeonbuk National Hospital. When the Xpert Xpress is run as a single assay for SARS-CoV-2, the test’s running time can be reduced to as little as 25 min because an early termination of the test can be employed (as per the package insert). In our study, the average TAT was about 5.8 min faster in the positive samples than in the negative samples. However, the BioFire COVID-19 Test and the RP 2.1, which was used as a multiplex assay, reported a running time of approximately 45 min, which was consistent with our examinations during this study [10]. Our study demonstrates that the BioFire COVID-19 Test, RP 2.1, and Cepheid Xpert Xpress have identical analytical performance to the reference SARS-CoV-2 RT-qPCR assay used for the detection of SARS-CoV-2. A comparative evaluation showed 100% positive and negative agreements between three simultaneous sample-to-answer SARS-CoV-2 RT-PCR assays in 77 individual NP swabs. Interestingly, the BioFire FilmArray and Cepheid Xpert Xpress systems showed the same analytical performance despite their completely different methodological principles. Even though the melting curve analysis using intercalating fluorescent dye that binds double-stranded DNA molecules between the DNA bases is not specific, and causes more false positives than qPCR using primers and a hydrolysis probe, which is often used for screening assays [17], the BioFire FilmArray overcomes the assay limitations by combining the multiplexing of the PCR and nesting together with the DNA-melting-curve analysis, to distinguish and identify multiple pathogens simultaneously [18].

In a previous report [8], the RP2.1 not only showed a comparable performance to high-throughput SARS-CoV-2 assays, such as the Hologic Panther Fusion and SARS-CoV-2 Roche Cobas SARS-CoV-2 assays, but also demonstrated comparable sensitivity to other sample-to-answer RT-PCR assays used for the identification of SARS-CoV-2. The RP2.1 is useful for identifying SARS-CoV-2 in recent infections, as well as in later phases of the disease, when viral loads drop significantly. Moreover, the RP2.1 detects 22 bacterial and viral respiratory pathogens including SARS-CoV-2, which allows for not only the identification of viral coinfection, but also the ability to simultaneously discriminate differential respiratory viral infections, such as seasonal influenza. A previous report suggested that the BioFire COVID-19 Test has a slightly reduced sensitivity compared to the Quanty COVID-19 RT-PCR system in COVID-19 diagnosis [12]. Other than the sample-to-answer principle, different target genes may cause additional false negatives. When considering which targets are the best candidates for SARS-CoV-2 assays, unique regions in genes should be considered. The BioFire COVID-19 Test uses *ORF1ab* and *ORF8* as targets within the SARS-CoV-2 genome. The *ORF1ab* target encodes 16 auto-proteolytically processed non-structural proteins, which generate the replicase–transcriptase complex. Host interactions with *ORF8* are associated with endomembrane compartments or vesicle-trafficking pathways, which may facilitate marked reconfigurations of endoplasmic reticulum and Golgi trafficking during coronavirus infection [19]. The *ORF1ab* and *N* regions are reported to be excellent targets for RT-qPCR assays due to their specificity for SARS-CoV-2 and their ability to create adequate PCR primers and probes for these regions [20]. This single-pathogen assay may prove helpful for pooled-sample testing, as resources become more limited during the pandemic, with regards to RP2.1 [6]. In emergency settings, pooled-sample testing is a very useful strategy to screen a large population cost-effectively with limited diagnostic resources. [21]. Although reference SARS-CoV-2 RT-qPCR assay could be applied as a pooled-sample testing strategy, sample-to-answer SARS-CoV-2 RT-PCR could be more helpful to generate rapid results because of its simple hands-on process. Unfortunately, the analytical performance of three sample-to-answer SARS-CoV-2 RT-PCR assays for a pooled-sample testing was not estimated in this study; a pool size of four or six is recommended when the Xpert Xpress or the RP 2.1 is used for a pooled-sample testing [22,23,24]. However, the medical laboratory should carefully validate the sample number for the pooled-sample testing to minimize false-negative results due to sample dilution before its implementation [21,24]

The Xpert Xpress showed an almost-perfect concordance with the high-throughput RT-qPCR batch platform such as Roche Cobas SARS-CoV-2 assay across the broad range of the tested Ct values, including rapid reporting with high viral loads, which could offer a shorter TAT [13,25]. The Xpert Xpress was reported as having one of the lowest limits of detection (100 copies/mL) compared to other sample-to-answer SARS-CoV-2 RT-PCR assays, such as the Abbott ID Now SARS-CoV-2, which possesses a proprietary isothermal nucleic acid amplification principle [26,27]. In particular, in the Xpert Xpress, an NP swab with a low viral titer, which is only positive for *E* gene repeatedly with a clear S-shaped amplification curve, could be regarded as positive for SARS-CoV-2 in regions where SARS-CoV-2 is prevalent, and in the absence of other widespread SARS-associated β coronaviruses (e.g., those leading to Middle East respiratory syndrome) [7]. However, our data showed that the Xpert Xpress exhibited prolonged Ct values for the *E* gene compared to the reference SARS-CoV-2 RT-qPCR assay. Medical laboratories are obligated to more carefully interpret the test results generated from the various sample-to-answer SARS-CoV-2 RT-PCR assays prior to implementation, particularly when very low viral loads are suspected, since positivity was observed at high Ct values [28]. Furthermore, they should carefully evaluate and monitor the performance of the sample-to-answer SARS-CoV-2 RT-PCR assays for rapid diagnosis, because the analytical performance of these sample-to-answer assays could be affected in a variety of situations [29,30]. Particularly, suboptimal analytical performance is associated with false-negative results in early or asymptomatic patients, and this can have a negative effect on prevention and control of nosocomial infections. Falasca and colleagues [28] reported several false-positive cases due to high Ct values of the *N2* gene only. In high prevalence regions, the high Ct values could indicate early, late, or remote infection, or may be false positive. When one targeted gene with high Ct values was detected only, the laboratory physician should be careful during interpretation and consider a review of the PCR amplification curves and analytical process [28]. In the Xpert Xpress, a result is interpreted as positive when only the *N2* gene is positive; however, a result is interpreted as presumptive positive when only the *E* gene is positive according to manufacturer’s instructions. Varadhan and colleagues [31] reported that samples that were only *N2* gene-positive with high Ct values over 40 were all false positive in their low prevalence condition. Khoshchehreh and colleagues [32] reported that samples that are the *N2* gene-positive but *E* gene-negative should be interpreted carefully, requiring an increase in the sensitivity of the Xpert Xpress in emergency settings. In their data, samples with only the *N2* gene detected made up 4% of all studied individuals; however, only 29.5% of patients were symptomatic among these. The asymptomatic individuals were younger than the symptomatic individuals. In the Korean guidelines for laboratory diagnosis of SARS-CoV-2, they recommend that a result is interpreted as positive when targeted genes are all positive, whereas re-testing or additional tests are required if only one of the multiple targets is positive [16]. Thus, to confirm the ambiguous SARS-CoV-2 results, two different principles of SARS-CoV-2 molecular assays should be operated, such as the reference SARS-CoV-2 RT-qPCR assay or the sample-to-answer SARS-CoV-2 RT-PCR assay.

On the other hand, the spread of the virus may be facilitated by a false-negative result, if these individuals remain capable of transmitting the virus. However, a false-negative result is less consequential if the presence of viral RNA in their samples reflects shedding at levels unlikely to result in transmission, or if it does not apply to the shedding of live virus [33,34]. Thus, when only one of the multiple target genes is positive, the prevalence of the virus in the patient’s region, their medical history, clinical manifestations, symptomatic features, and contact history should be considered comprehensively. Re-testing or alternative SARS-CoV-2 assay could be assessed to rule out false-positive results and to include early infection cases of COVID-19, and additional testing with follow-up samples could be considered. Samples with low viral loads are often obtained from individuals whose infections have clinically resolved or improved, but who need a negative test result to return to work, be released to another facility, or cease isolation measures [8]. Thus, in these cases, the past history should be required for a comprehensive interpretation.

There are some inherent limitations to our study, as the studied sample size was quite small, even though all of the samples showed concordant results between the sample-to-answer SARS-CoV-2 RT-PCR assays, compared to the reference SARS-CoV-2 RT-qPCR assay. Although rare, the possibility that the concordant SARS-CoV-2 results were false positives or false negatives cannot be ruled out, even though several comparative evaluations using small sample sizes (<100 clinical samples) were reported to be similar to our study [6,8,11,13]. Further studies with large sample sizes and wide ranges of CT values are required. Second, pre-analytical processes, including sample collection and preparation, infrastructure, and skilled personnel, which may have affected the results, were not estimated, even though the process between sample loading and interpretation of results is automated entirely when a sample-to-answer SARS-CoV-2 RT-PCR assay is applied. In particular, the risk of false positives and contamination is much higher when the sample-to-answer SARS-CoV-2 RT-PCR assay is conducted by non-laboratory-trained personnel and outside of a controlled test environment [27]. In addition, samples may have become too dilute in viral transport media, and low-level positives may have tested negative falsely [27]. Third, the samples tested in our study were collected in 2020 and did not include the Delta or Omicron variants. It has been reported that several point mutations, such as C29203T, C29200T, and C29197T may impair diagnostic sensitivity [35,36]. In particular, the single nucleotide variant (SNV) in the *N* gene, G29179T, was reported to influence the sensitivity of the Xpert Xpress [37]. The SNV of G29179T related to the B.1.497 is known to be most prevalent in the Republic of Korea, causing large differences between the Ct values of the *E* and *N2* genes and leading to significantly impaired diagnostic sensitivity. SARS-CoV-2 mutations and their viral variants pose great challenges to the accurate detection of SARS-CoV-2, as well as to vaccination processes and targeted viral therapy [38]. Thus, medical laboratories should be conscious of nucleotide variants in the SARS-CoV-2 genome and their potential impacts on the diagnosis of COVID-19, when the unusual pattern of Ct values is observed [27]. Fourth, the new Xpert Xpress CoV-2 plus test, which was recently launched in 2022, provides accurate and rapid results in as little as 20 min, offering faster sample-to-answer testing with actionable results from a single sample. More reliable virus detection will enable not only the optimization of N2 probes, but also three gene targets for SARS-CoV-2 [39].

## 5. Conclusions

In conclusion, our evaluation demonstrates that the BioFire COVID-19 Test, RP 2.1, and Cepheid Xpert Xpress assays compare favorably to the reference SARS-CoV-2 RT-qPCR assay, along with a 100% concordance in assay results for clinical samples and an acceptable analytical performance at their guaranteed limits of detection. The addition of a widely used simultaneous sample-to-answer SARS-CoV-2 RT-PCR assay will contribute to the number of medical laboratories able to test for COVID-19.

## Figures and Tables

**Table 1:** Performance characteristic information of the three sample-to-answer SARS-CoV-2 reverse transcription PCR assays provided by the manufacturer.

Characteristics	BioFire COVID-19 Test	BioFire Respiratory Panel 2.1	Xpert Xpress SARS-CoV-2
Manufacturer	BioFire Defense	BioFire Diagnostics	Cepheid
PCR equipment	BioFire FilmArray Torch Systems, BioFire FilmArray 2.0	BioFire FilmArray Torch Systems, BioFire FilmArray 2.0	GeneXpert Dx Instrument System
Principle of operation	DNA melting curve analysis	DNA melting curve analysis	Multiplex RT-PCR
Target genes	*ORF8* and *ORF1ab*	Membrane (*M*) and spike (*S*)	*N2* region of the nucleocapsid *(N)* and Envelope (*E*)
Limit of detection (copies/mL)	330	160 ^a^	250
Sample type	NPS	NPS	NPS, nasal wash/aspirate samples
Minimum sample volume (μL)	300	300	300
Running time of process (min)	50	50	45

RT-PCR, reverse transcription polymerase chain reaction; ORF, open reading frame; NPS, nasopharyngeal swab. ^a^ Obtained for culturing in a biosafety level 3 laboratory from the World Reference Center for Emerging Viruses and Arboviruses (WRCEVA), contributed by the U.S. Centers for Disease Control (CDC).

**Table 2:** Diagnostic performance statistics of BioFire COVID-19 Test, BioFire Respiratory Panel 2.1, and Cepheid Xpert Xpress SARS-CoV-2 assays for sample-to-answer detection in 77 clinical samples.

Statistics	BioFire COVID-19 Test	BioFire Respiratory Panel 2.1	Cepheid Xpert Xpress SARS-CoV-2
Kappa agreement	Almost perfect (Kappa, 1; 95% CI, 1 to 1)		
Concordance rate (%)	100	100	100
True positive (n)	36	36	36
True negative (n)	41	41	41
False positive (n)	0	0	0
False negative (n)	0	0	0
Sensitivity (%)	100 (95% CI, 90.26 to 100)	100 (95% CI, 90.26 to 100)	100 (95% CI, 90.26 to 100)
Specificity (%)	100 (95% CI, 91.40 to 100)	100 (95% CI, 91.40 to 100)	100 (95% CI, 91.40 to 100)
Positive likelihood ratio	Not available	Not available	Not available
Negative likelihood ratio	0 (95% CI, not available)	0 (95% CI, not available)	0 (95% CI, not available)
Percentage positive agreement * (%)	100 (95% CI, not available)	100 (95% CI, not available)	100 (95% CI, not available)
Percentage negative agreement * (%)	100 (95% CI, not available)	100 (95% CI, not available)	100 (95% CI, not available)
Accuracy * (%)	100 (95% CI, 95.32 to 100)	100 (95% CI, 95.32 to 100)	100 (95% CI, 95.32 to 100)

* In Republic of Korea, between 3 January 2020 and 23 December 2022, there had been 28,534,558 confirmed cases of COVID-19 and 31,674 deaths, as reported to the WHO. As of 26 October 2022, a total of 131,766,256 vaccine doses had been administered.

## Data Availability

Not applicable.
